# Outdated or underrated? The case for low-protein diets in CKD

**DOI:** 10.1590/2175-8239-JBN-2025-0065en

**Published:** 2025-07-04

**Authors:** Denise Mafra, Denis Fouque

**Affiliations:** 1 Universidade Federal Fluminense, Rio de Janeiro, RJ, Brazil.; 2 Université de Lyon, Department of Nephrology-Nutrition-Dialysis, Lyon, France.

Dear Dr. Miguel Riella,

We read the article entitled “**Protein restriction in CKD: an outdated strategy in the modern era**”^1^ with interest and would like to make some critical comments regarding its conclusions.

The article emphasizes that a low-protein diet (LPD), traditionally used in the management of non-dialysis CKD patients, has no proven efficacy in slowing the progression of the disease and could entail risks such as malnutrition and a decrease in quality of life. At the same time, the text suggests that the emphasis on new drugs would offer a more comprehensive strategy, with lifestyle changes taking a back seat.

The authors present old studies in Table 2, citing several unfounded criticisms. The MDRD study had flaws, including a too-short observation period and the inclusion of patients too early, before a 3-month steady state was achieved. Rosman et al.^2^ noted a delay in CKD progression with LPD and a reduction in serum urea, phosphate levels, and proteinuria. The criticisms, specifically the use of creatinine as an outcome measure and the subjective evaluation of LPD, do not invalidate the findings of this study. Such criticism appears unfounded when compared with the strong evidence demonstrating the benefits of the LPD approach^3^. Ihle et al.^4^ also found that LPD is effective in slowing CKD progression. Even slight differences in protein intake (0.69 vs. 0.85 g/kg/day) can significantly lower serum urea and phosphate levels, the main uremic toxins^5^. They also reported the significant weight loss in the intervention group. However, it is important to note that the diet did not change markers of muscle mass, such as arm circumference and triceps skinfold thickness.

Barsotti et al.^6^ wrote a letter to the editor emphasizing that inadequate adherence to the LPD identified in the Locatelli et al.^7^ study resulted in patients consuming significantly more protein than prescribed, undermining the intended LPD intervention. As reported in the study by Hansen et al.^8^, Bawazir et al.^1^ failed to identify a significant difference between groups in progression to CKD stage 5 or death, with 27% of patients on a normal protein diet compared to 10% on a LPD (P = 0.042). The relative risk of stage 5 CKD or death was 0.23 (0.07 to 0.72) for patients on LPD after baseline adjustment for the presence of cardiovascular disease (P = 0.01). It is worth noting that the so-called “most recent study” by Bawazir et al.^1^ is from 2009. Cianciaruso et al.^9^ concluded that a diet of 0.55 g/kg per day does not appear to offer an advantage in the survival of patients compared to the also low protein diet of 0.8 g/kg per day. Contrary to the claims made by Bawazir et al.^1^, the authors concluded that LPD does not increase the risk of malnutrition when a nutritionist is involved in the team.

Despite the controversy about protein restriction for CKD patients, more recent studies^10^ and meta-analyses^3^ highlight the substantial benefits of an LPD in slowing the progression of CKD, so much so that studies refer to the most recent nutrition guidelines^11^. Furthermore, the risk of eventual malnutrition can be minimized by proper nutritional monitoring, focusing on the quality of protein intake and energy intake, as recommended by experts in renal nutrition. Thus, the LPD remains the cornerstone of CKD treatment for non-dialysis patients. The diet should always be adapted to the individual needs of the patient to optimize results, considering factors such as CKD stage and the patient’s general health, age, nutritional status, and comorbidities^11^. It is not fair to patients to talk about quality of life without mentioning the tremendous loss of quality of life when patients start maintenance dialysis, as they can postpone the start of dialysis for many months or years by ingesting a LPD. For ethical reasons, this information should be provided to the patients, regardless of what doctors think.

When well-planned and monitored, protein restriction is a valuable tool and should not be labeled as “obsolete” without a more thorough discussion of each patient’s specific conditions, including comorbidities, stage of CKD, nutritional status, and current studies.

**Figure 1:**
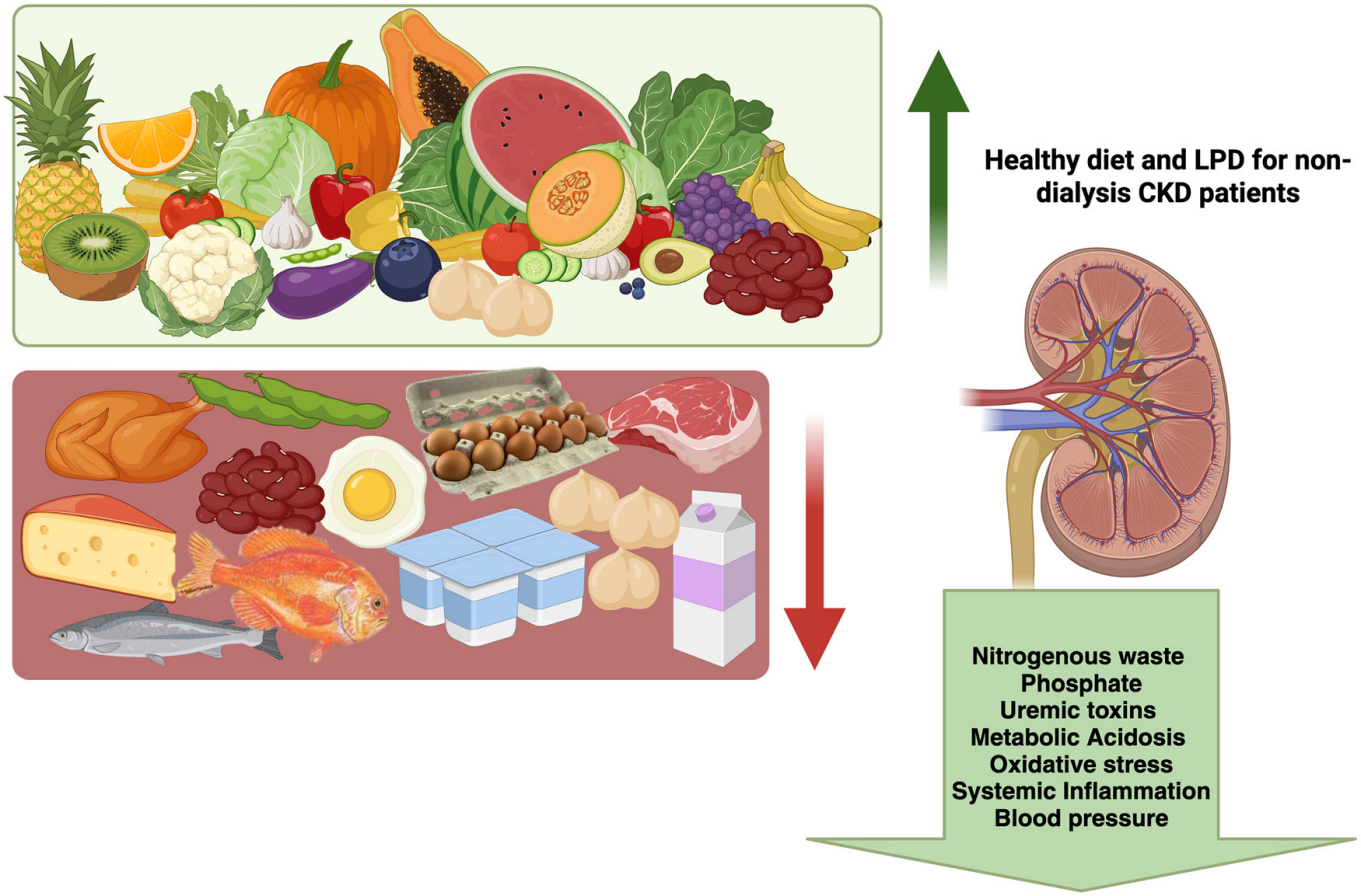
Low protein intake, when part of a healthy diet, can provide various benefits beyond slowing the progression of CKD. Created by BioRender^12^.

Moreover, a carefully monitored LPD offers several advantages beyond slowing the progression of CKD. By reducing protein intake and ensuring an adequate energy supply as part of a healthy diet, patients can experience less accumulation of nitrogenous waste, phosphate, and uremic toxins, which helps maintain acid-base balance and reduce inflammation and oxidative stress (Figure 1).

Presenting new medication options as the primary solution, implying that all dietary efforts are now worthless, is a gross misconception. It is crucial to be transparent about potential conflicts of interest and to clarify that while medications are valuable allies, they do not eliminate the need for a balanced and individualized approach to diet.

KDIGO and KDOQI guidelines emphasize that non-pharmacological interventions are crucial in reducing cardiovascular risk and the progression of CKD. The approach to CKD patients must be multidisciplinary and involve nephrologists, nutritionists, physical educators, psychologists, and nurses. Focusing the discussion exclusively on nephrologists and drugs may underestimate the complexity of CKD management.

Nutritional interventions combined with modern pharmacotherapy remain fundamental to the comprehensive care of CKD patients.
