# Supplementary material for: Outdated or underrated? The case for low-protein diets in CKD
Source: J Bras Nefrol. 2025 Jul 4;47(4):e20250065. doi: 10.1590/2175-8239-JBN-2025-0065en (PMC12233954; doi:10.1590/2175-8239-JBN-2025-0065en)
Supplement: Translation

# Translation: Obsoletas ou subestimadas? O caso das dietas hipoproteicas na DRC

**DOI:** 10.1590/2175-8239-JBN-2025-0065pt

**Authors:** Denise Mafra, Denis Fouque

**Affiliations:** 1 Universidade Federal Fluminense, Rio de Janeiro, RJ, Brasil.; 2 Université de Lyon, Department of Nephrology-Nutrition-Dialysis, Lyon, France.

Caro Dr. Miguel Riella,

Lemos o artigo intitulado “**Restrição proteica na DRC: uma estratégia obsoleta na era moderna**”^1^ com interesse e gostaríamos de fazer alguns comentários críticos a respeito de suas conclusões.

O artigo enfatiza que uma dieta de baixa proteína (LPD, do inglês *low protein diet*), tradicionalmente utilizada no manejo de pacientes com DRC não dialítica, não tem eficácia comprovada na desaceleração da progressão da doença e pode acarretar riscos como desnutrição e redução da qualidade de vida. Ao mesmo tempo, o texto sugere que os novos medicamentos oferecem estratégias mais abrangentes, deixando as mudanças dos hábitos alimentares e de vida num papel secundário.

Os autores apresentam estudos antigos na Tabela 2, mencionando diversas críticas infundadas. O estudo MDRD apresentou falhas, tais como o período de observação muito curto e a inclusão precoce de pacientes, antes que o estado estável de 3 meses fosse considerado. Rosman et al.^2^ observaram atraso na progressão da DRC com adoção da LPD e redução na ureia sérica, nos níveis de fosfato e na proteinúria. As críticas, incluindo creatinina como desfecho e a avaliação subjetiva da LPD, não invalidam os achados deste estudo. Essas críticas parecem infundadas quando comparadas com as evidências científicas que comprovam os benefícios da LPD^3^. Ihle et al.^4^ também constataram a eficácia da LPD em retardar a progressão da DRC. Mesmo pequenas diferenças na ingestão proteica (0,69 vs. 0,85 g/kg/dia) podem reduzir significativamente os níveis séricos de ureia e fosfato, principais toxinas urêmicas^5^. Eles também relataram a perda de peso significativa no grupo de intervenção. No entanto, é importante observar que a dieta não alterou os marcadores de massa muscular, como a circunferência do braço e a espessura da prega cutânea tricipital.

Barsotti et al.^6^ escreveram uma carta ao editor enfatizando que a adesão inadequada à LPD, identificada no estudo de Locatelli et al.^7^, resultou em pacientes consumindo significativamente mais proteína do que o prescrito, prejudicando a intervenção pretendida da dieta. Conforme relatado no estudo realizado por Hansen et al.^8^, Bawazir et al.^1^ não mostraram a diferença significativa entre grupos na progressão para o estágio 5 da DRC ou óbito, com 27% dos pacientes em uma dieta normoproteica comparados a 10% em LPD (P = 0,042). O risco relativo de DRC estágio 5 ou óbito foi de 0,23 (0,07 a 0,72) para pacientes em LPD, após o ajuste basal para a presença de doença cardiovascular (P = 0,01). Ressaltamos ainda que “estudo mais recente” apresentado por Bawazir et al.^1^ é de 2009. Cianciaruso et al.^9^ concluíram que uma dieta de 0,55 g/kg por dia não parece oferecer vantagem na sobrevida dos pacientes em comparação com uma dieta igualmente hipoproteica de 0,8 g/kg por dia. Contrariamente às alegações feitas por Bawazir et al.^1^, os autores concluíram que a LPD não aumenta o risco de desnutrição quando um nutricionista integra a equipe.

Apesar das controvérsias sobre a restrição proteica para pacientes com DRC, estudos^10^ e metanálises^3^ mais recentes destacam os benefícios substanciais de uma LPD para retardar a progressão da DRC, a ponto de os estudos referirem-se às diretrizes nutricionais mais recentes^11^. Além disso, o risco de eventual desnutrição pode ser minimizado por meio de monitoramento nutricional adequado, com foco na qualidade da ingestão de proteínas e na ingestão energética, conforme recomendado por especialistas em nutrição renal. Dessa forma, a LPD continua sendo a pedra angular do tratamento da DRC para pacientes não-dialíticos. A dieta deve sempre ser adaptada às necessidades individuais do paciente para otimizar os resultados, considerando fatores como o estágio da DRC e o estado geral de saúde, idade, estado nutricional e comorbidades do paciente^11^. Não é justo para os pacientes falar sobre qualidade de vida sem mencionar a enorme perda dessa qualidade quando eles iniciam a diálise, uma vez que é possível adiar o início da diálise por muitos meses ou anos com uma ingestão reduzida de proteínas. Por razões éticas, essas informações devem ser fornecidas aos pacientes, independentemente da opinião dos médicos.

Quando bem planejada e monitorada, a restrição proteica é uma ferramenta valiosa e não deve ser rotulada como “obsoleta” sem uma discussão mais aprofundada das condições específicas de cada indivíduo, incluindo comorbidades, estágio da DRC, estado nutricional e estudos atuais.

Além disso, uma LPD cuidadosamente monitorada oferece diversas vantagens que vão além do retardamento da progressão da DRC. Ao reduzir a ingestão de proteínas e garantir adequada ingestão de energia como parte de uma dieta saudável, os pacientes podem apresentar menor acúmulo de resíduos nitrogenados, fosfato e toxinas urêmicas, o que contribui para a manutenção do equilíbrio ácido-base e para a redução da inflamação e do estresse oxidativo (Figura 1).

**Figura 1:** A baixa ingestão de proteínas, quando parte de uma dieta saudável, pode proporcionar diversos benefícios além de retardar a progressão da DRC. Elaborada por BioRender^12^.

Apresentar novas opções de medicamentos como a principal solução, sugerindo que todos os esforços dietéticos se tornaram inúteis, é um grande equívoco. É fundamental que haja transparência em relação aos possíveis conflitos de interesse e que se esclareça que, embora os medicamentos sejam aliados valiosos, eles não eliminam a necessidade de uma abordagem equilibrada e individualizada da dieta.

As diretrizes do KDIGO e do KDOQI enfatizam que as intervenções não farmacológicas são cruciais para reduzir risco cardiovascular e progressão da DRC. A abordagem aos pacientes com DRC deve ser multidisciplinar e envolver nefrologistas, nutricionistas, educadores físicos, psicólogos e enfermeiros. Concentrar a discussão exclusivamente em nefrologistas e medicamentos pode subestimar a complexidade do manejo da DRC.

As intervenções nutricionais, combinadas à farmacoterapia moderna, permanecem fundamentais para o cuidado abrangente de pacientes com DRC.
